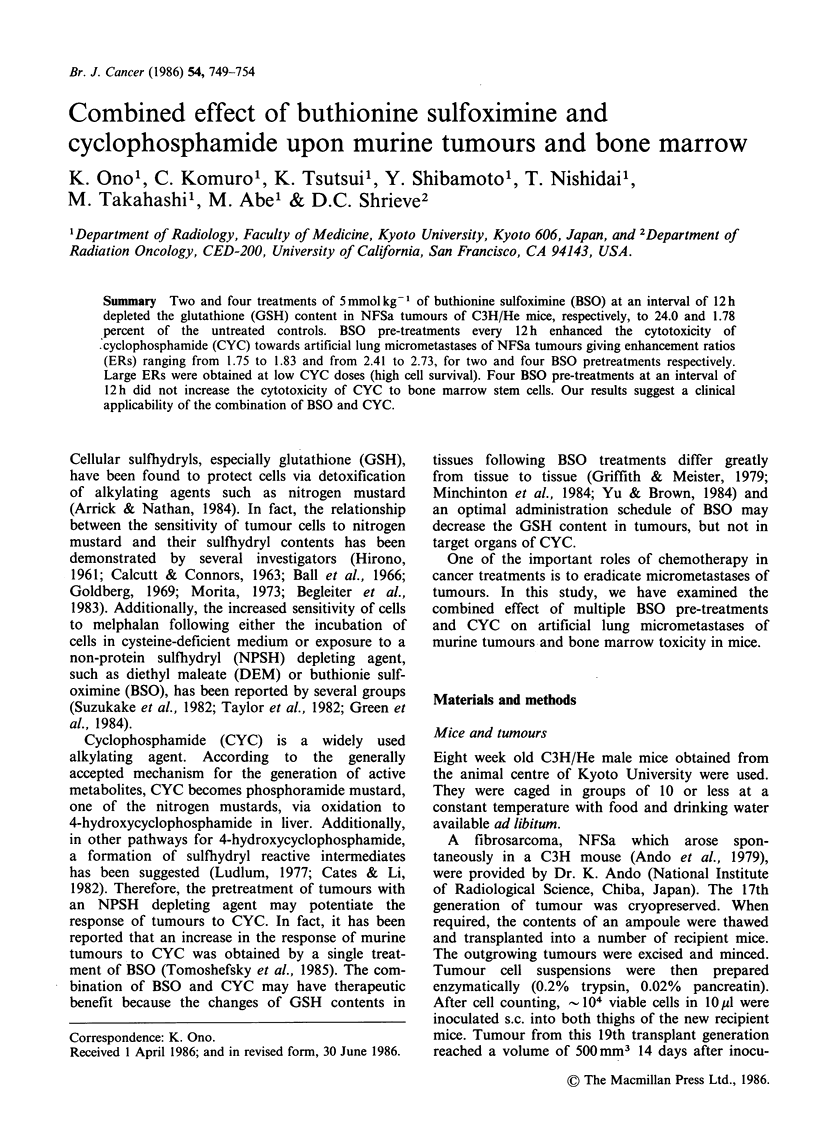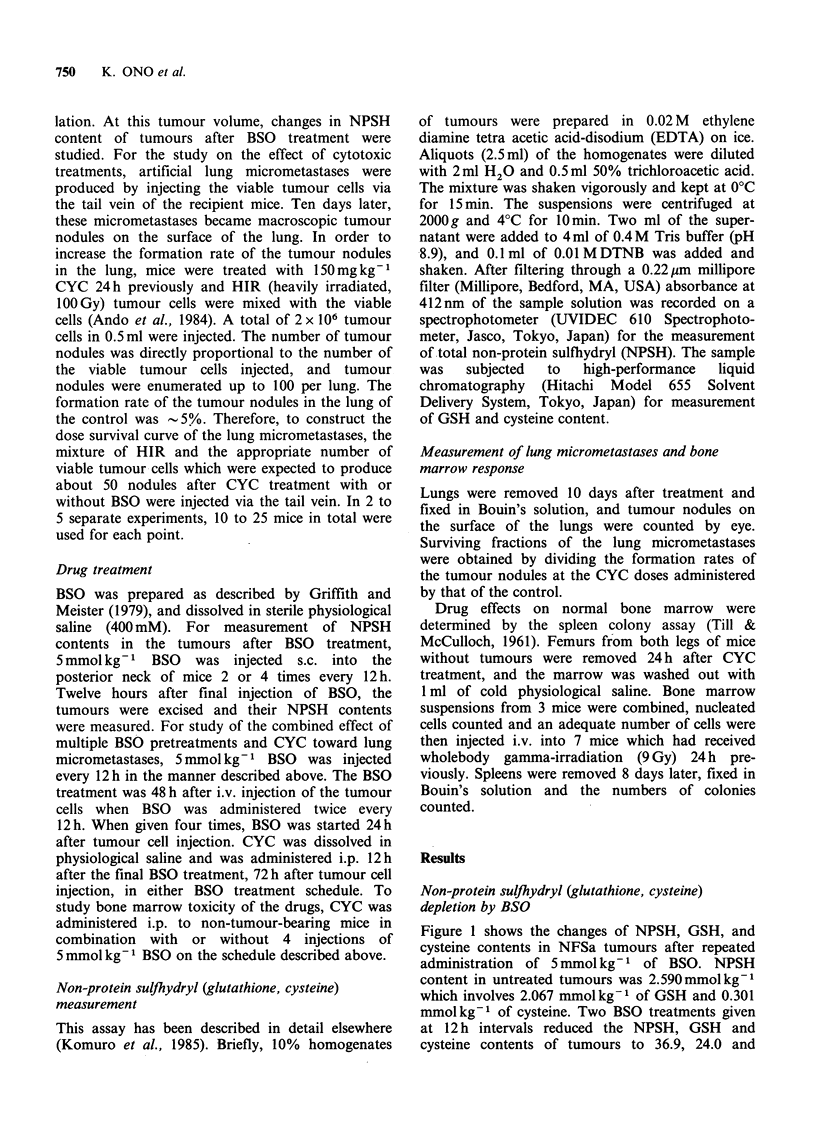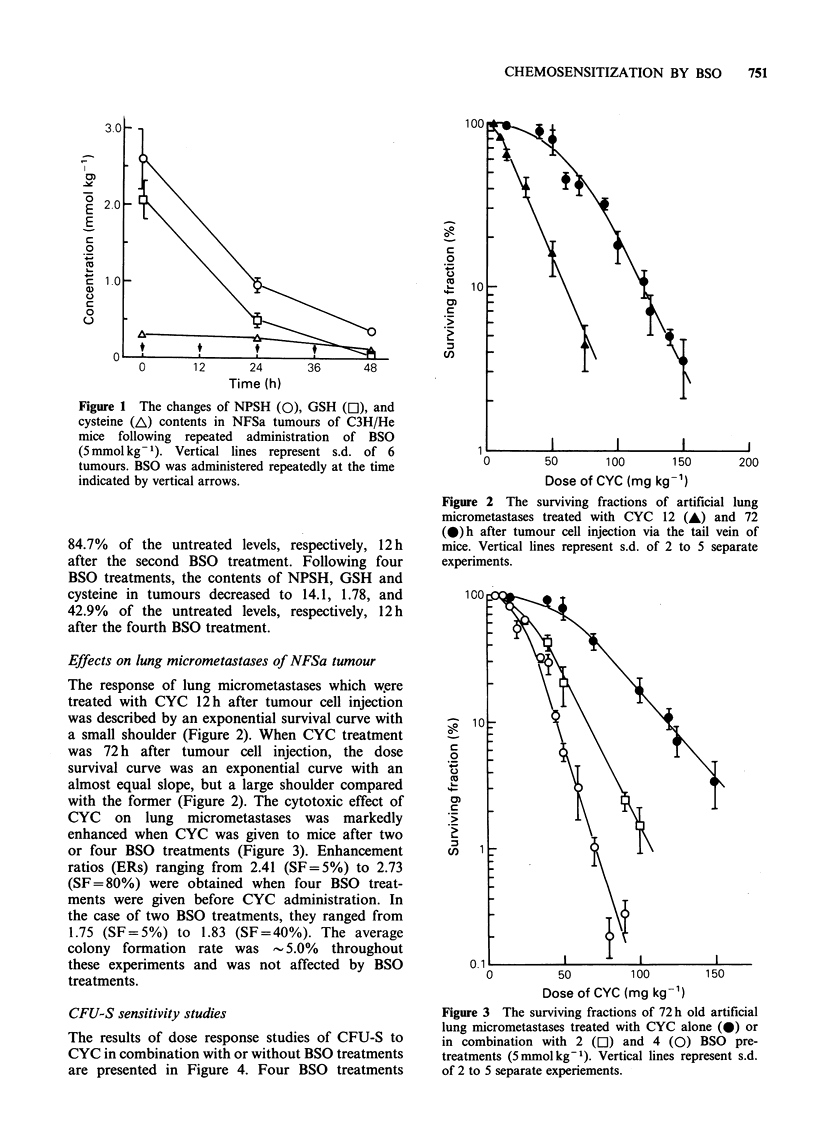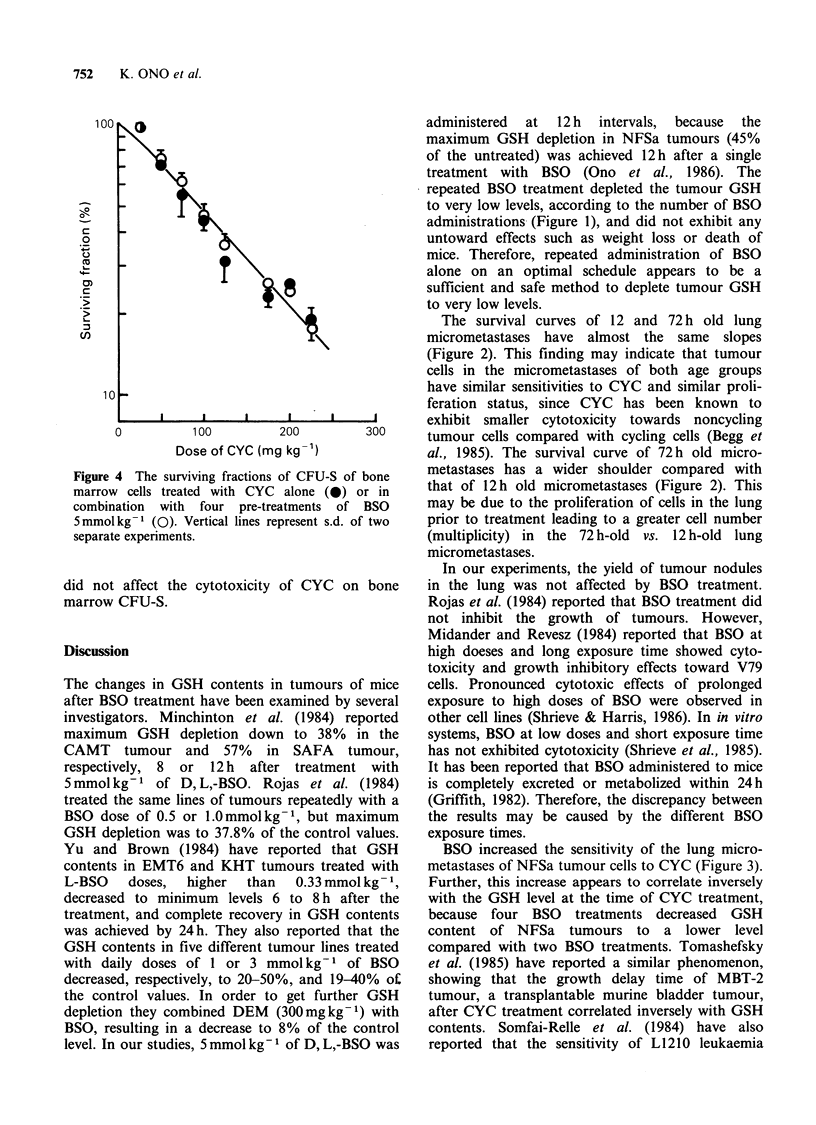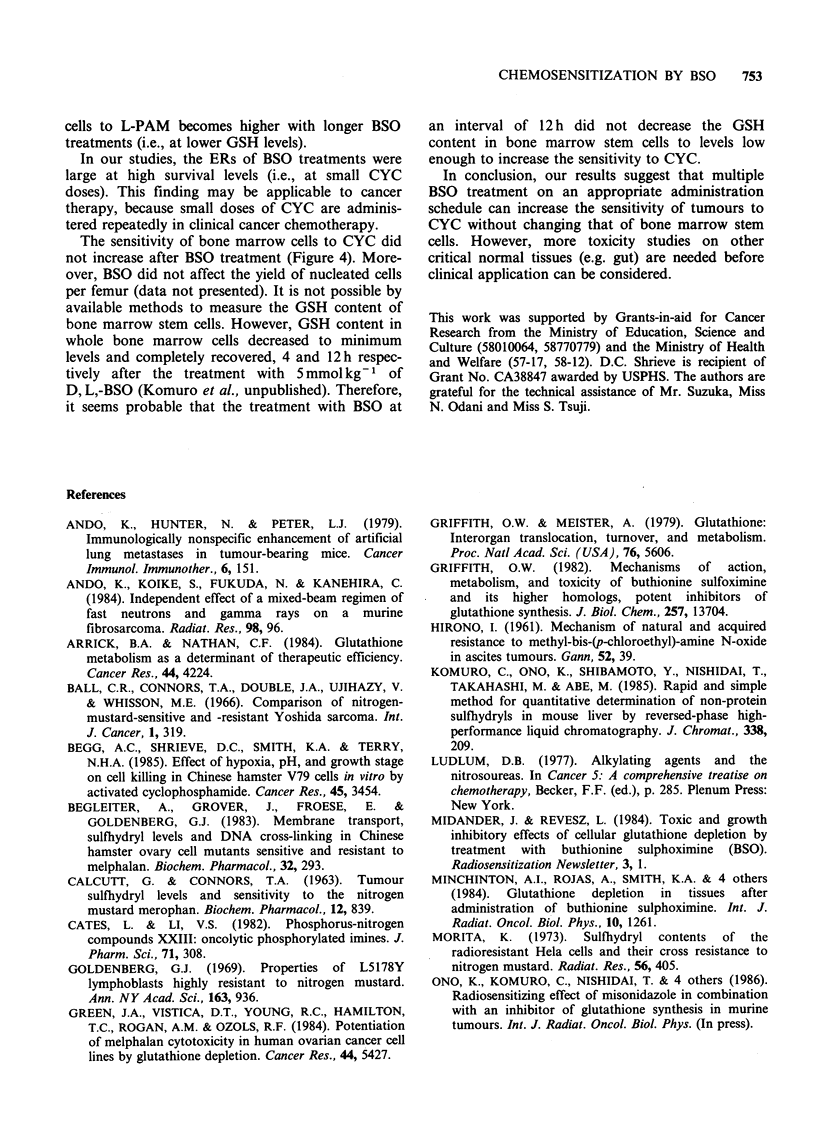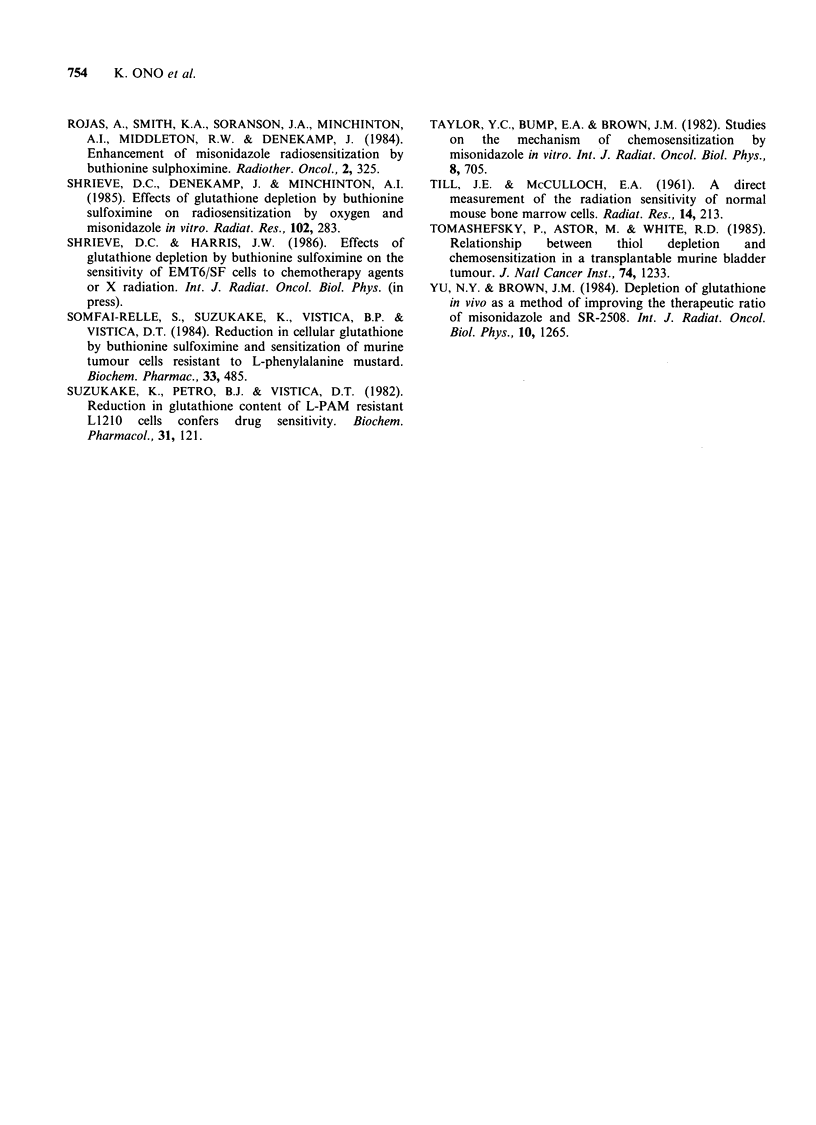# Combined effect of buthionine sulfoximine and cyclophosphamide upon murine tumours and bone marrow.

**DOI:** 10.1038/bjc.1986.236

**Published:** 1986-11

**Authors:** K. Ono, C. Komuro, K. Tsutsui, Y. Shibamoto, T. Nishidai, M. Takahashi, M. Abe, D. C. Shrieve

## Abstract

Two and four treatments of 5 mmol kg-1 of buthionine sulfoximine (BSO) at an interval of 12 h depleted the glutathione (GSH) content in NFSa tumours of C3H/He mice, respectively, to 24.0 and 1.78 percent of the untreated controls. BSO pre-treatments every 12 h enhanced the cytotoxicity of cyclophosphamide (CYC) towards artificial lung micrometastases of NFSa tumours giving enhancement ratios (ERs) ranging from 1.75 to 1.83 and from 2.41 to 2.73, for two and four BSO pretreatments respectively. Large ERs were obtained at low CYC doses (high cell survival). Four BSO pre-treatments at an interval of 12 h did not increase the cytotoxicity of CYC to bone marrow stem cells. Our results suggest a clinical applicability of the combination of BSO and CYC.


Br. J. Cancer (1986) 54, 749–754

### Combined effect of buthionine sulfoximine and cyclophosphamide upon murine tumours and bone marrow

K. Ono[1], C. Komuro[1], K. Tsutsui[1], Y. Shibamoto[1], T. Nishidai[1], M. Takahashi[1], M. Abe[1] & D.C. Shrieve[2]

[1]*Department of Radiology, Faculty of Medicine, Kyoto University, Kyoto 606, Japan, and* [2]*Department of Radiation Oncology, CED-200, University of California, San Francisco, CA 94143, USA.*

**Summary** Two and four treatments of 5 mmol kg$^{-1}$ of buthionine sulfoximine (BSO) at an interval of 12 h depleted the glutathione (GSH) content in NFSa tumours of C3H/He mice, respectively, to 24.0 and 1.78 percent of the untreated controls. BSO pre-treatments every 12 h enhanced the cytotoxicity of cyclophosphamide (CYC) towards artificial lung micrometastases of NFSa tumours giving enhancement ratios (ERs) ranging from 1.75 to 1.83 and from 2.41 to 2.73, for two and four BSO pretreatments respectively. Large ERs were obtained at low CYC doses (high cell survival). Four BSO pre-treatments at an interval of 12 h did not increase the cytotoxicity of CYC to bone marrow stem cells. Our results suggest a clinical applicability of the combination of BSO and CYC.

Cellular sulfhydryls, especially glutathione (GSH), have been found to protect cells via detoxification of alkylating agents such as nitrogen mustard (Arrick & Nathan, 1984). In fact, the relationship between the sensitivity of tumour cells to nitrogen mustard and their sulfhydryl contents has been demonstrated by several investigators (Hirono, 1961; Calcutt & Connors, 1963; Ball *et al.*, 1966; Goldberg, 1969; Morita, 1973; Begleiter *et al.*, 1983). Additionally, the increased sensitivity of cells to melphalan following either the incubation of cells in cysteine-deficient medium or exposure to a non-protein sulfhydryl (NPSH) depleting agent, such as diethyl maleate (DEM) or buthionie sulfoximine (BSO), has been reported by several groups (Suzukake *et al.*, 1982; Taylor *et al.*, 1982; Green *et al.*, 1984).

Cyclophosphamide (CYC) is a widely used alkylating agent. According to the generally accepted mechanism for the generation of active metabolites, CYC becomes phosphoramide mustard, one of the nitrogen mustards, via oxidation to 4-hydroxycyclophosphamide in liver. Additionally, in other pathways for 4-hydroxycyclophosphamide, a formation of sulfhydryl reactive intermediates has been suggested (Ludlum, 1977; Cates & Li, 1982). Therefore, the pretreatment of tumours with an NPSH depleting agent may potentiate the response of tumours to CYC. In fact, it has been reported that an increase in the response of murine tumours to CYC was obtained by a single treatment of BSO (Tomoshefsky *et al.*, 1985). The combination of BSO and CYC may have therapeutic benefit because the changes of GSH contents in tissues following BSO treatments differ greatly from tissue to tissue (Griffith & Meister, 1979; Minchinton *et al.*, 1984; Yu & Brown, 1984) and an optimal administration schedule of BSO may decrease the GSH content in tumours, but not in target organs of CYC.

One of the important roles of chemotherapy in cancer treatments is to eradicate micrometastases of tumours. In this study, we have examined the combined effect of multiple BSO pre-treatments and CYC on artificial lung micrometastases of murine tumours and bone marrow toxicity in mice.

### Materials and methods

### *Mice and tumours*

Eight week old C3H/He male mice obtained from the animal centre of Kyoto University were used. They were caged in groups of 10 or less at a constant temperature with food and drinking water available *ad libitum*.

A fibrosarcoma, NFSa which arose spontaneously in a C3H mouse (Ando *et al.*, 1979), were provided by Dr. K. Ando (National Institute of Radiological Science, Chiba, Japan). The 17th generation of tumour was cryopreserved. When required, the contents of an ampoule were thawed and transplanted into a number of recipient mice. The outgrowing tumours were excised and minced. Tumour cell suspensions were then prepared enzymatically (0.2% trypsin, 0.02% pancreatin). After cell counting, $\sim 10^4$ viable cells in 10 $\mu$l were inoculated s.c. into both thighs of the new recipient mice. Tumour from this 19th transplant generation reached a volume of 500 mm$^3$ 14 days after inocu-

Correspondence: K. Ono.
Received 1 April 1986; and in revised form, 30 June 1986.

© The Macmillan Press Ltd., 1986.

lation. At this tumour volume, changes in NPSH content of tumours after BSO treatment were studied. For the study on the effect of cytotoxic treatments, artificial lung micrometastases were produced by injecting the viable tumour cells via the tail vein of the recipient mice. Ten days later, these micrometastases became macroscopic tumour nodules on the surface of the lung. In order to increase the formation rate of the tumour nodules in the lung, mice were treated with $150\,mg\,kg^{-1}$ CYC 24 h previously and HIR (heavily irradiated, 100 Gy) tumour cells were mixed with the viable cells (Ando *et al.*, 1984). A total of $2 \times 10^6$ tumour cells in 0.5 ml were injected. The number of tumour nodules was directly proportional to the number of the viable tumour cells injected, and tumour nodules were enumerated up to 100 per lung. The formation rate of the tumour nodules in the lung of the control was ~5%. Therefore, to construct the dose survival curve of the lung micrometastases, the mixture of HIR and the appropriate number of viable tumour cells which were expected to produce about 50 nodules after CYC treatment with or without BSO were injected via the tail vein. In 2 to 5 separate experiments, 10 to 25 mice in total were used for each point.

*Drug treatment*

BSO was prepared as described by Griffith and Meister (1979), and dissolved in sterile physiological saline (400 mM). For measurement of NPSH contents in the tumours after BSO treatment, $5\,mmol\,kg^{-1}$ BSO was injected s.c. into the posterior neck of mice 2 or 4 times every 12 h. Twelve hours after final injection of BSO, the tumours were excised and their NPSH contents were measured. For study of the combined effect of multiple BSO pretreatments and CYC toward lung micrometastases, $5\,mmol\,kg^{-1}$ BSO was injected every 12 h in the manner described above. The BSO treatment was 48 h after i.v. injection of the tumour cells when BSO was administered twice every 12 h. When given four times, BSO was started 24 h after tumour cell injection. CYC was dissolved in physiological saline and was administered i.p. 12 h after the final BSO treatment, 72 h after tumour cell injection, in either BSO treatment schedule. To study bone marrow toxicity of the drugs, CYC was administered i.p. to non-tumour-bearing mice in combination with or without 4 injections of $5\,mmol\,kg^{-1}$ BSO on the schedule described above.

*Non-protein sulfhydryl (glutathione, cysteine) measurement*

This assay has been described in detail elsewhere (Komuro *et al.*, 1985). Briefly, 10% homogenates of tumours were prepared in 0.02 M ethylene diamine tetra acetic acid-disodium (EDTA) on ice. Aliquots (2.5 ml) of the homogenates were diluted with 2 ml $H_2O$ and 0.5 ml 50% trichloroacetic acid. The mixture was shaken vigorously and kept at 0°C for 15 min. The suspensions were centrifuged at 2000 *g* and 4°C for 10 min. Two ml of the supernatant were added to 4 ml of 0.4 M Tris buffer (pH 8.9), and 0.1 ml of 0.01 M DTNB was added and shaken. After filtering through a 0.22 $\mu$m millipore filter (Millipore, Bedford, MA, USA) absorbance at 412 nm of the sample solution was recorded on a spectrophotometer (UVIDEC 610 Spectrophotometer, Jasco, Tokyo, Japan) for the measurement of total non-protein sulfhydryl (NPSH). The sample was subjected to high-performance liquid chromatography (Hitachi Model 655 Solvent Delivery System, Tokyo, Japan) for measurement of GSH and cysteine content.

*Measurement of lung micrometastases and bone marrow response*

Lungs were removed 10 days after treatment and fixed in Bouin's solution, and tumour nodules on the surface of the lungs were counted by eye. Surviving fractions of the lung micrometastases were obtained by dividing the formation rates of the tumour nodules at the CYC doses administered by that of the control.

Drug effects on normal bone marrow were determined by the spleen colony assay (Till & McCulloch, 1961). Femurs from both legs of mice without tumours were removed 24 h after CYC treatment, and the marrow was washed out with 1 ml of cold physiological saline. Bone marrow suspensions from 3 mice were combined, nucleated cells counted and an adequate number of cells were then injected i.v. into 7 mice which had received wholebody gamma-irradiation (9 Gy) 24 h previously. Spleens were removed 8 days later, fixed in Bouin's solution and the numbers of colonies counted.

### Results

*Non-protein sulfhydryl (glutathione, cysteine) depletion by BSO*

Figure 1 shows the changes of NPSH, GSH, and cysteine contents in NFSa tumours after repeated administration of $5\,mmol\,kg^{-1}$ of BSO. NPSH content in untreated tumours was $2.590\,mmol\,kg^{-1}$ which involves $2.067\,mmol\,kg^{-1}$ of GSH and 0.301 $mmol\,kg^{-1}$ of cysteine. Two BSO treatments given at 12 h intervals reduced the NPSH, GSH and cysteine contents of tumours to 36.9, 24.0 and

**Figure 1** The changes of NPSH (○), GSH (□), and cysteine (△) contents in NFSa tumours of C3H/He mice following repeated administration of BSO (5 mmol kg$^{-1}$). Vertical lines represent s.d. of 6 tumours. BSO was administered repeatedly at the time indicated by vertical arrows.

**Figure 2** The surviving fractions of artificial lung micrometastases treated with CYC 12 (▲) and 72 (●) h after tumour cell injection via the tail vein of mice. Vertical lines represent s.d. of 2 to 5 separate experiments.

84.7% of the untreated levels, respectively, 12 h after the second BSO treatment. Following four BSO treatments, the contents of NPSH, GSH and cysteine in tumours decreased to 14.1, 1.78, and 42.9% of the untreated levels, respectively, 12 h after the fourth BSO treatment.

### *Effects on lung micrometastases of NFSa tumour*

The response of lung micrometastases which were treated with CYC 12 h after tumour cell injection was described by an exponential survival curve with a small shoulder (Figure 2). When CYC treatment was 72 h after tumour cell injection, the dose survival curve was an exponential curve with an almost equal slope, but a large shoulder compared with the former (Figure 2). The cytotoxic effect of CYC on lung micrometastases was markedly enhanced when CYC was given to mice after two or four BSO treatments (Figure 3). Enhancement ratios (ERs) ranging from 2.41 (SF = 5%) to 2.73 (SF = 80%) were obtained when four BSO treatments were given before CYC administration. In the case of two BSO treatments, they ranged from 1.75 (SF = 5%) to 1.83 (SF = 40%). The average colony formation rate was ~5.0% throughout these experiments and was not affected by BSO treatments.

**Figure 3** The surviving fractions of 72 h old artificial lung micrometastases treated with CYC alone (●) or in combination with 2 (□) and 4 (○) BSO pretreatments (5 mmol kg$^{-1}$). Vertical lines represent s.d. of 2 to 5 separate experiements.

### *CFU-S sensitivity studies*

The results of dose response studies of CFU-S to CYC in combination with or without BSO treatments are presented in Figure 4. Four BSO treatments

**Figure 4** The surviving fractions of CFU-S of bone marrow cells treated with CYC alone (●) or in combination with four pre-treatments of BSO 5 mmol kg$^{-1}$ (○). Vertical lines represent s.d. of two separate experiments.

did not affect the cytotoxicity of CYC on bone marrow CFU-S.

### Discussion

The changes in GSH contents in tumours of mice after BSO treatment have been examined by several investigators. Minchinton *et al.* (1984) reported maximum GSH depletion down to 38% in the CAMT tumour and 57% in SAFA tumour, respectively, 8 or 12 h after treatment with 5 mmol kg$^{-1}$ of D, L,-BSO. Rojas *et al.* (1984) treated the same lines of tumours repeatedly with a BSO dose of 0.5 or 1.0 mmol kg$^{-1}$, but maximum GSH depletion was to 37.8% of the control values. Yu and Brown (1984) have reported that GSH contents in EMT6 and KHT tumours treated with L-BSO doses, higher than 0.33 mmol kg$^{-1}$, decreased to minimum levels 6 to 8 h after the treatment, and complete recovery in GSH contents was achieved by 24 h. They also reported that the GSH contents in five different tumour lines treated with daily doses of 1 or 3 mmol kg$^{-1}$ of BSO decreased, respectively, to 20–50%, and 19–40% of the control values. In order to get further GSH depletion they combined DEM (300 mg kg$^{-1}$) with BSO, resulting in a decrease to 8% of the control level. In our studies, 5 mmol kg$^{-1}$ of D, L,-BSO was administered at 12 h intervals, because the maximum GSH depletion in NFSa tumours (45% of the untreated) was achieved 12 h after a single treatment with BSO (Ono *et al.*, 1986). The repeated BSO treatment depleted the tumour GSH to very low levels, according to the number of BSO administrations (Figure 1), and did not exhibit any untoward effects such as weight loss or death of mice. Therefore, repeated administration of BSO alone on an optimal schedule appears to be a sufficient and safe method to deplete tumour GSH to very low levels.

The survival curves of 12 and 72 h old lung micrometastases have almost the same slopes (Figure 2). This finding may indicate that tumour cells in the micrometastases of both age groups have similar sensitivities to CYC and similar proliferation status, since CYC has been known to exhibit smaller cytotoxicity towards noncycling tumour cells compared with cycling cells (Begg *et al.*, 1985). The survival curve of 72 h old micrometastases has a wider shoulder compared with that of 12 h old micrometastases (Figure 2). This may be due to the proliferation of cells in the lung prior to treatment leading to a greater cell number (multiplicity) in the 72 h-old *vs.* 12 h-old lung micrometastases.

In our experiments, the yield of tumour nodules in the lung was not affected by BSO treatment. Rojas *et al.* (1984) reported that BSO treatment did not inhibit the growth of tumours. However, Midander and Revesz (1984) reported that BSO at high doeses and long exposure time showed cytotoxicity and growth inhibitory effects toward V79 cells. Pronounced cytotoxic effects of prolonged exposure to high doses of BSO were observed in other cell lines (Shrieve & Harris, 1986). In *in vitro* systems, BSO at low doses and short exposure time has not exhibited cytotoxicity (Shrieve *et al.*, 1985). It has been reported that BSO administered to mice is completely excreted or metabolized within 24 h (Griffith, 1982). Therefore, the discrepancy between the results may be caused by the different BSO exposure times.

BSO increased the sensitivity of the lung micrometastases of NFSa tumour cells to CYC (Figure 3). Further, this increase appears to correlate inversely with the GSH level at the time of CYC treatment, because four BSO treatments decreased GSH content of NFSa tumours to a lower level compared with two BSO treatments. Tomashefsky *et al.* (1985) have reported a similar phenomenon, showing that the growth delay time of MBT-2 tumour, a transplantable murine bladder tumour, after CYC treatment correlated inversely with GSH contents. Somfai-Relle *et al.* (1984) have also reported that the sensitivity of L1210 leukaemia cells to L-PAM becomes higher with longer BSO treatments (i.e., at lower GSH levels).

In our studies, the ERs of BSO treatments were large at high survival levels (i.e., at small CYC doses). This finding may be applicable to cancer therapy, because small doses of CYC are administered repeatedly in clinical cancer chemotherapy.

The sensitivity of bone marrow cells to CYC did not increase after BSO treatment (Figure 4). Moreover, BSO did not affect the yield of nucleated cells per femur (data not presented). It is not possible by available methods to measure the GSH content of bone marrow stem cells. However, GSH content in whole bone marrow cells decreased to minimum levels and completely recovered, 4 and 12 h respectively after the treatment with 5 $mmol\,kg^{-1}$ of D, L,-BSO (Komuro *et al.*, unpublished). Therefore, it seems probable that the treatment with BSO at an interval of 12 h did not decrease the GSH content in bone marrow stem cells to levels low enough to increase the sensitivity to CYC.

In conclusion, our results suggest that multiple BSO treatment on an appropriate administration schedule can increase the sensitivity of tumours to CYC without changing that of bone marrow stem cells. However, more toxicity studies on other critical normal tissues (e.g. gut) are needed before clinical application can be considered.

This work was supported by Grants-in-aid for Cancer Research from the Ministry of Education, Science and Culture (58010064, 58770779) and the Ministry of Health and Welfare (57-17, 58-12). D.C. Shrieve is recipient of Grant No. CA38847 awarded by USPHS. The authors are grateful for the technical assistance of Mr. Suzuka, Miss N. Odani and Miss S. Tsuji.

### References

ANDO, K., HUNTER, N. & PETER, L.J. (1979). Immunologically nonspecific enhancement of artificial lung metastases in tumour-bearing mice. *Cancer Immunol. Immunother.*, **6**, 151.

ANDO, K., KOIKE, S., FUKUDA, N. & KANEHIRA, C. (1984). Independent effect of a mixed-beam regimen of fast neutrons and gamma rays on a murine fibrosarcoma. *Radiat. Res.*, **98**, 96.

ARRICK, B.A. & NATHAN, C.F. (1984). Glutathione metabolism as a determinant of therapeutic efficiency. *Cancer Res.*, **44**, 4224.

BALL, C.R., CONNORS, T.A., DOUBLE, J.A., UJIHAZY, V. & WHISSON, M.E. (1966). Comparison of nitrogen-mustard-sensitive and -resistant Yoshida sarcoma. *Int. J. Cancer*, **1**, 319.

BEGG, A.C., SHRIEVE, D.C., SMITH, K.A. & TERRY, N.H.A. (1985). Effect of hypoxia, pH, and growth stage on cell killing in Chinese hamster V79 cells *in vitro* by activated cyclophosphamide. *Cancer Res.*, **45**, 3454.

BEGLEITER, A., GROVER, J., FROESE, E. & GOLDENBERG, G.J. (1983). Membrane transport, sulfhydryl levels and DNA cross-linking in Chinese hamster ovary cell mutants sensitive and resistant to melphalan. *Biochem. Pharmacol.*, **32**, 293.

CALCUTT, G. & CONNORS, T.A. (1963). Tumour sulfhydryl levels and sensitivity to the nitrogen mustard merophan. *Biochem. Pharmacol.*, **12**, 839.

CATES, L. & LI, V.S. (1982). Phosphorus-nitrogen compounds XXIII: oncolytic phosphorylated imines. *J. Pharm. Sci.*, **71**, 308.

GOLDENBERG, G.J. (1969). Properties of L5178Y lymphoblasts highly resistant to nitrogen mustard. *Ann. NY Acad. Sci.*, **163**, 936.

GREEN, J.A., VISTICA, D.T., YOUNG, R.C., HAMILTON, T.C., ROGAN, A.M. & OZOLS, R.F. (1984). Potentiation of melphalan cytotoxicity in human ovarian cancer cell lines by glutathione depletion. *Cancer Res.*, **44**, 5427.

GRIFFITH, O.W. & MEISTER, A. (1979). Glutathione: Interorgan translocation, turnover, and metabolism. *Proc. Natl Acad. Sci. (USA)*, **76**, 5606.

GRIFFITH, O.W. (1982). Mechanisms of action, metabolism, and toxicity of buthionine sulfoximine and its higher homologs, potent inhibitors of glutathione synthesis. *J. Biol. Chem.*, **257**, 13704.

HIRONO, I. (1961). Mechanism of natural and acquired resistance to methyl-bis-(*p*-chloroethyl)-amine N-oxide in ascites tumours. *Gann*, **52**, 39.

KOMURO, C., ONO, K., SHIBAMOTO, Y., NISHIDAI, T., TAKAHASHI, M. & ABE, M. (1985). Rapid and simple method for quantitative determination of non-protein sulfhydryls in mouse liver by reversed-phase high-performance liquid chromatography. *J. Chromat.*, **338**, 209.

LUDLUM, D.B. (1977). Alkylating agents and the nitrosoureas. In *Cancer 5: A comprehensive treatise on chemotherapy*, Becker, F.F. (ed.), p. 285. Plenum Press: New York.

MIDANDER, J. & REVESZ, L. (1984). Toxic and growth inhibitory effects of cellular glutathione depletion by treatment with buthionine sulphoximine (BSO). *Radiosensitization Newsletter*, **3**, 1.

MINCHINTON, A.I., ROJAS, A., SMITH, K.A. & 4 others (1984). Glutathione depletion in tissues after administration of buthionine sulphoximine. *Int. J. Radiat. Oncol. Biol. Phys.*, **10**, 1261.

MORITA, K. (1973). Sulfhydryl contents of the radioresistant Hela cells and their cross resistance to nitrogen mustard. *Radiat. Res.*, **56**, 405.

ONO, K., KOMURO, C., NISHIDAI, T. & 4 others (1986). Radiosensitizing effect of misonidazole in combination with an inhibitor of glutathione synthesis in murine tumours. *Int. J. Radiat. Oncol. Biol. Phys.* (In press).

ROJAS, A., SMITH, K.A., SORANSON, J.A., MINCHINTON, A.I., MIDDLETON, R.W. & DENEKAMP, J. (1984). Enhancement of misonidazole radiosensitization by buthionine sulphoximine. *Radiother. Oncol.*, **2,** 325.

SHRIEVE, D.C., DENEKAMP, J. & MINCHINTON, A.I. (1985). Effects of glutathione depletion by buthionine sulfoximine on radiosensitization by oxygen and misonidazole *in vitro*. *Radiat. Res.*, **102,** 283.

SHRIEVE, D.C. & HARRIS, J.W. (1986). Effects of glutathione depletion by buthionine sulfoximine on the sensitivity of EMT6/SF cells to chemotherapy agents or X radiation. *Int. J. Radiat. Oncol. Biol. Phys.* (in press).

SOMFAI-RELLE, S., SUZUKAKE, K., VISTICA, B.P. & VISTICA, D.T. (1984). Reduction in cellular glutathione by buthionine sulfoximine and sensitization of murine tumour cells resistant to L-phenylalanine mustard. *Biochem. Pharmac.*, **33,** 485.

SUZUKAKE, K., PETRO, B.J. & VISTICA, D.T. (1982). Reduction in glutathione content of L-PAM resistant L1210 cells confers drug sensitivity. *Biochem. Pharmacol.*, **31,** 121.

TAYLOR, Y.C., BUMP, E.A. & BROWN, J.M. (1982). Studies on the mechanism of chemosensitization by misonidazole *in vitro*. *Int. J. Radiat. Oncol. Biol. Phys.*, **8,** 705.

TILL, J.E. & McCULLOCH, E.A. (1961). A direct measurement of the radiation sensitivity of normal mouse bone marrow cells. *Radiat. Res.*, **14,** 213.

TOMASHEFSKY, P., ASTOR, M. & WHITE, R.D. (1985). Relationship between thiol depletion and chemosensitization in a transplantable murine bladder tumour. *J. Natl Cancer Inst.*, **74,** 1233.

YU, N.Y. & BROWN, J.M. (1984). Depletion of glutathione *in vivo* as a method of improving the therapeutic ratio of misonidazole and SR-2508. *Int. J. Radiat. Oncol. Biol. Phys.*, **10,** 1265.